# Theory-based formative research on oral rehydration salts and zinc use in Lusaka, Zambia

**DOI:** 10.1186/s12889-016-2984-2

**Published:** 2016-04-12

**Authors:** Katie Greenland, Jenala Chipungu, Roma Chilengi, Valerie Curtis

**Affiliations:** Department of Disease Control, Faculty for Infectious and Tropical Diseases, London School of Hygiene & Tropical Medicine, Keppel Street, London, WC1E 7HT UK; Centre for Infectious Disease Research in Zambia, P.O. Box 34681, Lusaka, 10101 Zambia

**Keywords:** Formative research, Oral rehydration, Zinc, Diarrhoea prevention, Intervention design, Behaviour centred design

## Abstract

**Background:**

A theoretically grounded formative research study was carried out to investigate behaviour related to the use of Oral Rehydration Salts (ORS) and zinc tablets. The purpose was to inform the design of the behaviour change component of the Programme for Awareness and Elimination of Diarrhoea in Lusaka Province, Zambia, which aims to reduce childhood morbidity and mortality from diarrhoeal disease.

**Methods:**

Fourteen behaviour trials were conducted among caregivers of children under-five with diarrhoea. Caregivers were recruited from two clinics situated in rural and peri-urban Lusaka. Trials took ten days and data were captured using video, observation and repeated interviews. Additional data were collected through focus group discussions with mothers, observations in clinics and pharmacies and interviews with clinic and pharmacy staff. Findings were organised according to categories of behavioural determinants from Evo-Eco theory.

**Results:**

Participants were all familiar with ORS and most knew its purpose. ORS use was motivated by symptoms of dehydration, rather than the start of a diarrhoea episode, and was stopped when the child had visibly recovered energy. Only four of 14 behaviour trial participants were observed to correctly prepare ORS. Errors were mainly associated with measurement, resulting in a solution that was too concentrated. ORS was not observed to be given to children at clinics. Although zinc was unknown in this population, it was positively received by mothers keen to learn whether zinc would work better than alternative treatments to stop diarrhoea.

**Conclusions:**

ORS was sub-optimally prepared and used at home. It was not used while waiting to be seen at a clinic. In homes, the behaviour change intervention should promote early and continued use of correctly prepared ORS. In the longer-term, these behaviours may best be encouraged by changing the product design or sachet size. Despite its unfamiliarity, this population was well disposed to the use of zinc as a treatment for diarrhoea; when zinc is new to a population, promoting zinc as a solution to stopping diarrhoea, which mothers seek, may drive initial trial. Ensuring the availability of zinc in public clinics and private pharmacies prior to commencement of any promotion activities is crucial.

## Background

Despite declines in overall child mortality, as well as in diarrhoeal deaths over the last 10 years [1, 2], diarrhoea remains the second leading cause of child deaths globally [2]. It was estimated to cause over half a million (0·448–0·750 million) deaths among children under-five in 2013 [2]. Though oral rehydration salts (ORS) are known to prevent and treat dehydration, the major cause of death from diarrhoea, their use is far from ubiquitous [3]. ORS has been available since the 1970s, when its initial introduction was accompanied by intense promotion efforts [4, 5], but funding and interest in diarrhoea control subsequently waned. Despite renewed attention and the development of more effective, low-osmolarity ORS in the early 2000s [6], ORS use remains low, and has even declined in many countries [7], with only a third of childhood diarrhoeal episodes estimated to receive ORS in sub-Saharan Africa [6].

More recently, zinc supplementation has been shown to be an important complement to ORS, reducing the duration and severity of diarrhoeal episodes [8]. A complete 10–14 day course of zinc supplement can also reduce subsequent incidence of diarrhoea [8]. WHO and UNICEF have recommended zinc supplementation and ORS for the routine management of acute childhood diarrhoea since 2004 [9]. Zinc has been introduced via the private sector and included on the ‘essential drugs’ list in the Integrated Management of Childhood Illnesses (IMCI) package in many countries. However, it is still not procured in sufficient quantities to meet needs, and is not widely purchased and sold in the private sector [6, 10]. Whilst it has been estimated that universal coverage and use of ORS and zinc to treat diarrhoea could prevent three-quarters of diarrhoea-associated mortality [11] and promotion of zinc may help to drive ORS uptake [9], there is limited information about how best to promote ORS and zinc.

In Zambia, the risk of a child dying before their fifth birthday is high (75 deaths per 1000 live births), with diarrhoea the cause of 1 in 5 of these deaths [12]. Sixty-four percent of diarrhoeal episodes among children under five are reported to receive ORS [12], but Zinc use is negligible. Despite higher reported use than elsewhere in the region, a ‘LisT’ analysis placed the promotion of ORS third on the list of interventions that could save the most child lives in Zambia [13].

The Programme for Awareness and Elimination of Diarrhoea (PAED) aims to reduce childhood morbidity and mortality from diarrhoeal disease through strategies including the use of ORS and zinc for the home management of child diarrhoea in Lusaka Province. A formative research study was carried out to inform the design of the ORS and zinc promotion component of the programme.

**Fig. 1:**
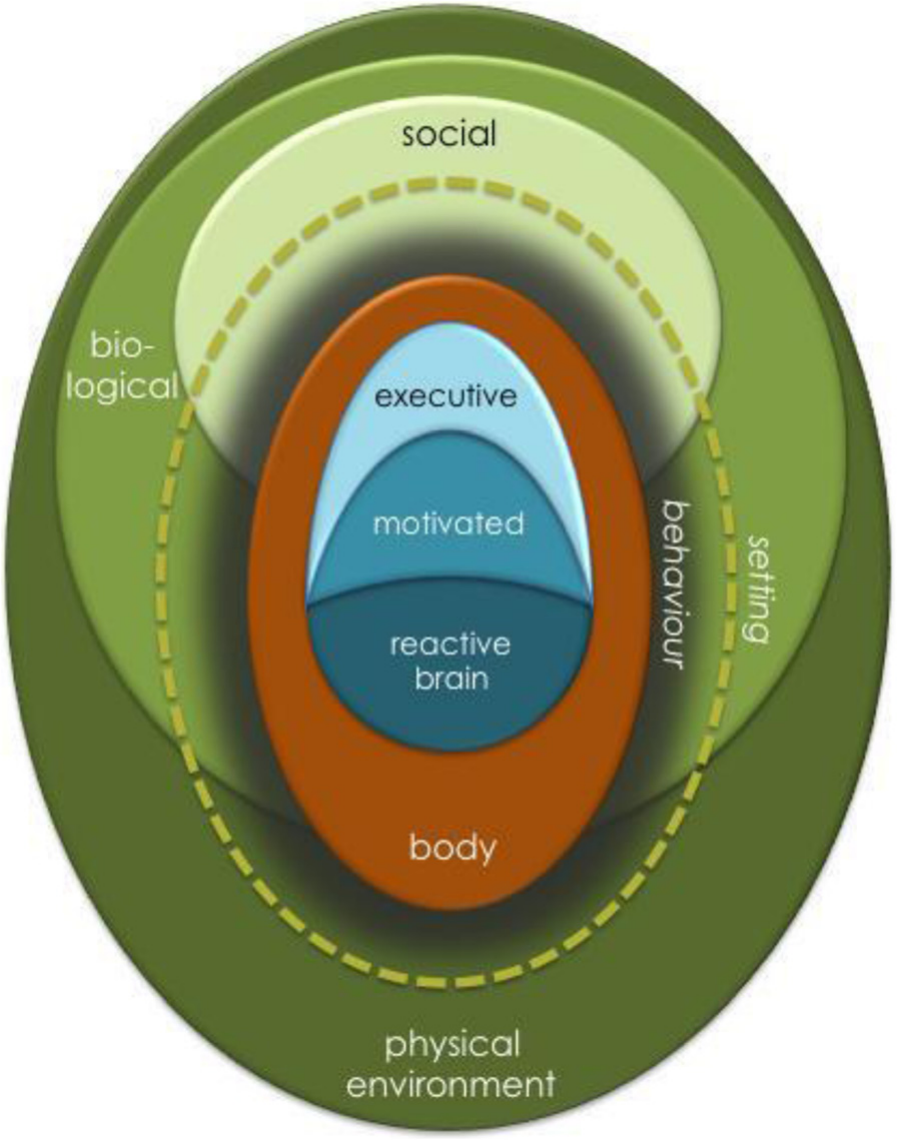
Evo-Eco model of the determinants of behaviour [15]

This work was guided by the ‘Behaviour Centred Design’ (BCD) approach [14], a process for designing behaviour change interventions. Central to this is the Evo-Eco model of behavioural determinants (Fig. 1) [15]. The model proposes that the brain controls behaviour in three major ways: some actions are automatic responses to cues; others are driven by a motive such as fear or affiliation to reach particular evolved goal states such as safety or social acceptance; and others originate from conscious, cognitive decisions that result from internal evaluation of the – often longer-term - consequences of different courses of action [16]. A person’s actions are also influenced by aspects of their physical, social and biological environment which determine most routine and recurring behaviours – the ‘setting’, in which the behaviour takes place [17].

The BCD approach also outlines five essential steps for the design and testing of behaviour change interventions: ***A****ssess* what is known and what is not known about the behavioural problem; ***B****uild* on this understanding by carrying out formative research; ***C****reate* a theory-based intervention; ***D****eliver* it; and ***E****valuate* it [14, 18]. This paper concerns the *Assess* and *Build* steps for ORS and zinc promotion for home management of childhood diarrhoea in Lusaka, Zambia. Other aspects of the formative research are described by Chipungu et al., (In prep). Findings from the evaluation of the intervention developed as a result of this research will appear elsewhere.

## Methods

### Study setting and population

The study was conducted in Lusaka Province, Zambia, which is comprised of four districts and is home to just over 2 million of Zambia’s 13 million inhabitants [19]. Eighty percent of the province’s population reside in Lusaka District, the majority in peri-urban slums known as ‘compounds’. These areas are characterised by high population density and poor access to water supply, electricity, roads, waste management and sanitation. The remaining three districts in Lusaka Province are rural and sparsely populated, with houses more geographically distant from health centres and other facilities.

The main target of the behaviour change campaign was envisaged to be primary caregivers of children under-five in peri-urban and rural settings. Accordingly, two research sites were selected in a peri-urban compound in Lusaka District and in a rural area in Chongwe District.

### Assess: Framing the research questions

Following the BCD process, the first step was to assess what was known and not known about use of ORS and zinc in home management of diarrhoea to determine the parameters of the formative research. A framing workshop was convened to review evidence from published journal articles, grey literature and expert knowledge on the practice of the target behaviours and their determinants in Zambia and elsewhere. Though some reports on clinic prescribing practices and reported use of ORS from household surveys were located, little was known about how caregivers prepared and used ORS to manage diarrhoea at home. The Assess stage also established that the supply of zinc via government health centres and private pharmacies (drug stores) was unreliable. Three objectives for the formative research were set:

1. Define current behaviour and what precisely needs to be changed (target behaviour) and by whom (target audiences).
2. Investigate the determinants of current practices and the context in which they occur, in order to identify how change may be brought about.
3. Explore how people communicate currently to identify channels that could be used to reach the target audience (*not described in this paper*)*.*

### Build: Formative research methods

In keeping with our theoretical focus on behaviour, the research concentrated on documenting behaviour and its unconscious drivers rather than on what people said that they thought, did or believed. Consequently, data were primarily collected through observation of behaviour in context and behaviour trials [20]. Data were collected iteratively until researchers were satisfied that all topics had been adequately covered.

### Behaviour trials

The clinical management of diarrhoea in Zambia has a standard protocol: children with no dehydration are put on ‘Plan A’ and sent home with ORS, zinc, and any other necessary medications; moderately-dehydrated children are put on ‘Plan B’ and are observed receiving ORS at an oral rehydration therapy (ORT) corner before they are reassessed; and children that are more severely dehydrated are admitted and receive intravenous fluid, known as ‘Plan C’ [21]. A convenience sample of caregivers with a child under five with diarrhoea on ‘Plan A’ treatment were recruited after they had collected their medications at the pharmacy in the government clinics in the two study sites. Zinc was provided to the pharmacies to ensure that recruited mothers would receive a full and correct course.

The behaviour trial involved three separate home visits during a 10 day period. The first visit was on the day of recruitment. The purpose of the study was explained to the participant, written informed consent was obtained and they were accompanied home from the clinic. They were then interviewed about the child’s diarrhoea and were observed and videoed as they prepared ORS solution using the sachet they had been given by the clinic. Corrections were made as necessary at the end of the visit. Participants were provided with three additional sachets of ORS and were encouraged to buy more (to be reimbursed) if needed. The ill child was observed taking the first zinc tablet during this visit. Information on the purpose of zinc and how it should be administered (once daily) was provided and the participant was left with a calendar as a daily reminder to give zinc. Two further visits were made, once between the 3^rd^ and 6^th^ day after recruitment and again on the 9^th^ or 10^th^ day. Participants were interviewed on each visit using a topic guide and the presence of ORS and number of remaining zinc tablets were documented. All interviews were voice-recorded.

### Focus group discussions and key informant interviews

Four focus group discussions were conducted in both study sites with female primary caregivers of children under-five not participating in the behaviour trial. Up to 10 caregivers were identified by a Neighbourhood Health Committee volunteer; group size was restricted to encourage participation. Projective techniques [22] were used to explore motives behind decisions to give ORS and zinc by inviting the participants to select cards depicting different human motives to explain how a comic strip story of a child with diarrhoea could best be concluded. Picture-sorting exercises were also used to explore how ORS and zinc were classified and understood in the context of other treatments. A probing topic guide was used to direct discussion to cover common practices and perceptions about diarrhoea, its causes and impact on the mother, and knowledge and opinions of available traditional and biomedical treatment of diarrhoea.

### Clinic and pharmacy visits

Unstructured observations were carried out over 2–3 h in clinic waiting rooms and ORT corners in both clinics during recruitment for behaviour trials. Private pharmacies (drug stores) in the vicinity of the clinics were visited. Short, semi-structured interviews were conducted with three health workers at public and private health clinics and two pharmacy shop workers to understand caregiver treatment-seeking practices and provide insight into provider behaviour.

### Piloting and training

Data collection tools were field tested and revised prior to and during a week of intensive classroom and practical training. By the end of training all team members were knowledgeable about ORS and zinc and understood how to observe behaviour and probe appropriately during interviews and discussions.

### Data handling and analysis

The videos of ORS preparation were reviewed and a summary narrative was produced. ORS preparation was divided into four stages according to guidelines for preparation [23] and instructions for use on ORS sachets in Zambia: 1) use of safe water, i.e. chlorinated water or water that has been boiled; 2) measurement of 1 l of water, measured after boiling, if applicable; 3) use of the whole sachet of ORS, put into 1 l of cooled water (if applicable); and 4) the prepared ORS covered appropriately to prevent contamination. The summary narratives were then used to ascertain which stages of preparation were associated with error. All audio recordings of interviews and focus group discussions were transcribed. QSR International’s *Nvivo* 10 qualitative data analysis software was used to organise transcripts and to code segments of interviews into nodes related to use of ORS or zinc based *a priori* on the ‘Evo-Eco’ determinants of behaviour. This coding was used to identify emergent determinants related to the use and preparation of ORS and the use of zinc.

## Results

### Characteristics of study participants

Fourteen caregivers in the peri-urban (*n* = 8) and rural (*n* = 6) study areas participated in behaviour trials between February and March 2013. All were the mothers of the ill child with the exception of one grandmother. Two of the individuals approached declined to participate, stating that they were not returning home after the clinic. Socio-demographic characteristics of participants are presented in Table 1. Four focus groups were also held with groups of 7–10 mothers of children under-five in the peri-urban (*n* = 2) and rural (*n* = 2) communities.

**Table 1:** Socio-demographic profile of behaviour trial participants (*N* = 14)

ID	Age of mother (years)	Age of ill child (months)	Marital status	Occupation	Occupation of main breadwinner	Household monthly income (ZMK)	Mother's education level	HH size
GEORGE (*n* = 8)								
1	46–50*	54–59	Married	Self-employed	General worker	500-100	Primary	8
2	21–25	12–17	Single	Unemployed	Unemployed	Unknown	Primary	11
3	21–25	18–23	Divorced	Unemployed		<500	Primary	14
4	26–30	18–23	Married	Self-employed	Manual labourer	<500	None	6
5	21–25	24–29	Married	Self-employed	Businessman	500-1000	Primary	4
6	26–30	6–11	Divorced	Self-employed		500-1000	None	8
7	16–20	0–5	Married	Student	Businessman	>1000	Some secondary	3
8	21–25	18–23	Married	Unemployed	Salaried job	500-1000	Some secondary	3
NGWERERE (*N* = 6)								
1	21–25	12–17	Married	Self-employed	Businessman	<500	Secondary	5
2	21–25	60	Divorced	Unemployed	Businessman	<500	Some secondary	3
3	21–25	12–17	Single	Unemployed	Agriculture	<500	None	5
4	26–30	12–17	Married	Unemployed	Manual labourer	500-1000	Primary	4
5	21–25	30–35	Married	Unemployed	Businessman	500-1000	Some secondary	4
6	26–30	18–23	Divorced	Self-employed		<500	Primary	3

*Caregiver is the child’s grandmother

### Clinic characteristics

Clinics in both study sites were clean at the time of our unscheduled visits, and had functioning toilets and space to wait within the clinic and its surroundings. Many patients had gathered before the clinics opened and the peri-urban clinic was especially crowded. Waiting times were several hours in both clinics. Queues were shorter in rural clinics, but there were fewer attending staff.

Despite dozens of cases of diarrhoea passing through the clinics during the period of observation, the ORT corners at both clinics remained unsupervised and unused. The clinic pharmacies had ORS but only one or two sachets were given per queueing patient. Courses of 10 zinc tablets were cut in half in the rural clinic; reportedly because of limited supplies. Staff admitted failing to write a prescription for zinc as it is not always available for purchase elsewhere. Two private pharmacies in the vicinity of the peri-urban clinic had small quantities of ORS and zinc, but sales staff (who were not qualified pharmacists) could neither correctly describe how to prepare ORS, nor knew the dosage and function of zinc. No pharmacies were found near the rural clinic.

### Caregiver behaviour

Findings concerning the three behaviours of interest are presented below: 1) ORS use; 2) ORS preparation; and 3) zinc use by caregivers. The behaviours are first described, followed by the possible determinants of current practices. The latter are organised according to the categories in the Evo-Eco model of behaviour and are summarised in Table 2.

**Table 2:** Factors influencing use of ORS and zinc in the management of childhood diarrhoea by theoretical construct

	Insufficient early and continuous usage of ORS	Incorrect preparation of ORS	Trial of zinc
FACTORS IN THE BRAIN			
Executive * Deliberate planning, knowledge*	Awareness of ORS and its purpose to replace fluids is well-*understood* - caregivers know that it does not stop diarrhoea	Limited *knowledge* of the importance of preparing correctly-concentrated solution as ORS is considered to work like rice water or an energy drink	Zinc is *unknown* and thus not part of current suite of medicines considered to treat diarrhoea.
	Some *knowledge* that ORS should be given from onset of diarrhoea but lack of knowledge that it should be given throughout the episode	High level of *knowledge* regarding the use of clean water for ORS preparation but limited knowledge that 1 packet of ORS needs to be prepared with 1 l of water	*Belief* that injections/antibiotics are the best way of stopping diarrhoea
	*Planning:* ORS may not be stocked at home ready to use		*Planning*: Zinc is not kept at home so is unavailable as a choice of treatment at start of diarrhoea episode
Motivated * Emotional drivers, interests, reward*	It is *rewarding* when children respond to ORS (energy, play, eat). When a child is visibly sick (listless, dehydrated) mothers respond with ORS	*Hoard:* small volumes of ORS deliberately prepared using part sachets to avoid wasting limited supplies or preparing ORS that is not consumed by the child	*Nurture:* eager to stop diarrhoea in their child (and other family members with diarrhoea)
	*Nurture:* Mothers primarily seek treatment to stop diarrhoea so ORS is not highly valued	*Affiliation:* eager to learn how to prepare ORS correctly for social approval	*Curiosity:* keen to see whether zinc is as 'strong' as other diarrhoea medicines
	ORS is given away or is sold cheaply so is not highly *valued*	*Hunger/Thirst,* ORS given more easily when child complains of thirst	*Hunger:* Zinc tastes unpleasant but children could be persuaded to take it.
Reactive * Cues, habits & skills*	ORS initiation is *cued* in response to dehydration rather than at onset of each diarrhoeal episode and discontinued when child visibly recovers	*Cue* to stop adding ORS sachet contents to water is when it ‘tastes right’	Reminders were often needed to *cue* daily zinc administration and to give zinc for 10 days
FACTORS IN THE ENVIRONMENT			
Physical * Objects/tools, infrastructure*	Few ORS *sachets* are given at clinic, limiting ability to give ORS throughout diarrhoeal episode without purchasing more or returning to the clinic	Many households lack a *container* to measure 1 l of water. ‘Banana cups’ no longer found frequently	Zinc in *blister packs* or unmarked plastic bags look like common painkillers
	It takes appreciable time and charcoal to boil water on a *brazier* that is needed for multiple purposes	*Sachets* to make up smaller quantities of ORS are not available	
	Caregivers move around during the day and carrying ORS *solution* can be awkward	Water is hauled from *standpipes* and stored in *containers* in the house	
Social * Role models, relationships, networks, norms, institutions*	Waiting time to get prescription and then get ORS dispensed at *clinic* is high	*Neighbours* may lend ORS and give previously used or left over medications	Lack of accountability for giving zinc when shared with multiple *family members*
	Lack of private *pharmacies* in rural areas	*Clinics* give only one or two sachets of ORS, often after queuing for a long period	People talk. They share experiences of remedies that work and trial them.
	Older (female) *relatives and neighbours* advise on appropriate treatment		Clinic *pharmacies* are the main source of medicine, but zinc is often unavailable and may not be given/prescribed. Lack of availability in private sector in rural areas even if affordable.
	Illness may be concealed because people can *gossip* about why a child is ill		Clinic staff are trusted to determine the correct treatment
Biological * Parasites/foods/* *animals*	*Diarrhoea* is a common phenomenon	Home environment is hard to keep free of *contamination* (lack of running water/hard surfaces, cleaning equipment, toilets, etc.)	

#### Behaviour 1: Insufficient early initiation and continued use of ORS

ORS was often given at home soon after a child had fallen ill with diarrhoea, but use of ORS was not always initiated at the onset of diarrhoea and did not continue until the end of the episode as recommended.

During the time between onset of diarrhoea and presentation at the clinic up to five days later, 10 of the 14 behaviour trial participants claimed to have given their child homemade sugar-salt solution (*n* = 4) or manufactured ORS (*n* = 6) that they had at home or had borrowed from a neighbour. Other medicines, including paracetamol (*n* = 6), metronidazole antibiotic and antiprotazoal medication (Flagyl™) (*n* = 3) and anti-malaria medications (*n* = 1) were also given at home before treatment was sought from a clinic. Use of traditional remedies was only reported by one trial participant, although further discussion in focus groups indicated that their use is quite common in these communities:

*‘We cause a lot of deaths because we like to start with this same Bemba style [traditional medicine]. It is rare for us to rush first to the clinic’ (Focus group participant, Peri-urban).*

ORS solution or half-empty sachets were found in three homes during the first visit conducted immediately after recruitment. Six children were still ill with diarrhoea at the time of the first follow up visit; ORS solution was found in just one of these households.

### Factors influencing Behaviour 1: Insufficient early initiation and continued use of ORS

#### Factors in the Brain

At the executive level of behavioural determinants, *knowledge* of ORS use was high. In particular, all caregivers had heard of ORS and almost all understood that it does not stop diarrhoea.

‘W*hen the child has diarrhoea, the child loses water, so when you prepare this [ORS], you can then give the child as many times as possible.... So that the water in the body can be replenished’ (Mother, Peri-urban).*

Most *knew* that it should be given before going to the clinic and *believed* that ORS restored energy in a [dehydrated] child, from the experience of seeing the effects on their own or other children (all caregivers had used ORS previously). Few *knew* that ORS solution should be given throughout the course of the episode. Few mothers *planned* for possible diarrhoea by keeping a stock of ORS ready in the home, or *intended* to give the child ORS regularly until the diarrhoea stopped.

At the level of motivation, the ‘*nurture’* motive emerged as an important driver of action; caregivers worried about their ill children and acted accordingly:

*‘When I was taking him to the clinic, he scared me. Every time I looked at him, he was quiet. When I would carry him, he could not even cry.’ (Mother, Rural).*

Their primary reason for seeking treatment at the clinic was to stop the diarrhoea, and mothers reported feeling frustrated when they were given only ORS. Nevertheless, ORS, which is known locally as *‘manzi yamoyo’* (water of life), was valued for use when a child was ill and lacked energy to eat or play. Mothers’ motivation to continue ORS once a child had visibly recovered energy, however, was low:

*‘Even just after the child has finished this amount, the child will have enough water in the body.... Well, because of the way the child appears, if the child looks energetic and the body seems to have regained its form, you can then stop’ (Mother, Rural).*

Hence, though mothers *knew* that ORS should be given from the onset of diarrhoea, in practice they responded with ORS only when a child was thirsty, crying and visibly suffering, signs of which *cued* a nurturing response. Once these signs stopped, ORS use stopped also.

#### Factors in the Environment

*Physical* factors in the environment that constrained the early initiation of ORS use were its lack of immediate availability and the physical difficulties of preparing it. ORS was not usually present at home and a visit to a clinic often required an early start and change of plans for the day. Once ORS had been acquired, often after a long wait, preparation was also time-consuming as water was generally boiled and cooled. This further delayed receipt of ORS by the ill child.

This conversation with one peri-urban mother illustrates the issues:

Researcher: *‘Have you made fresh ORS?’* Participant: *‘No not yet. I have just finished cooking rice so the brazier is now free and I can boil the water. Now, he kept saying amu amu, so I figured he was hungry and opted to cook the rice first.’* Researcher (later in the conversation): *‘Why have you not made any ORS up to this time?’* Participant: *‘I don’t have boiled water’.* Researcher: *‘Are you boiling some now?’* Participant: *‘No, I am boiling some for tea first.’* Researcher: *‘How do you usually make it? Do you get water from the water that you are boiling for tea or do you boil the water for ORS separately?’* Participant: *I wait for the school-going children to finish cooking their food first then I boil the water for ORS.’*

Once initiated, running out of ORS sachets constrained the continued use of ORS, even in this study where three sachets were provided:

*‘I saw that she had some energy so I thought if I make any more it will get spoilt… The clinic doesn’t give any [ORS], so this one will help later on.’ (Mother, Peri-urban).*

Early initiation and continued use was also influenced by *social* factors. Mothers disliked having to reveal that their child was ill, fearing gossip and stigma, hence hoped that an episode would self-limit and not have to be acknowledged publically in a clinic visit.

‘*People like to gossip… Some would say I neglect the child and that is what is causing the child to get diarrhoea’ (Mother, Peri-urban).*

Eight of the 14 participants lived with a mother or mother-in-law. These older women recommended use of ORS when a child shows symptoms of dehydration:

*‘My mother is the one that used to tell [when the child needed ORS], she would just say I am an elderly woman, I know… [Researcher: What did she do?] She was checking the child’s eyes and hands.’ (Mother, Rural).*

Diarrhoea was seen as an inevitable fact of life that occurs often, for example in association with teething, and which resolves spontaneously. Together these factors conspired to desensitise mothers to the potential severity of diarrhoea, a common *biological* factor in the environment.

#### Behaviour 2: Incorrect preparation of ORS

Video data showed that ORS was prepared sub-optimally (Table 3) with errors at all stages.

**Table 3:** ORS preparation by behaviour trial participants (*N* = 14)

ID	Summary of observed ORS preparation	Prepared using clean water?	1 l of water measured?	Whole sachet added, into cool water?	Solution covered?	ORS prepared correctly according to all criteria?
GEORGE						
1	Took a bowl, washed jug in the bowl, took water from stored (chlorinated) water using a banana cup (500 ml) and measured two cupfuls into the jug. Mixed in whole sachet of ORS with spoon. Poured ORS into a kettle and covered with bowl.	Yes	Yes	Yes	Yes	Yes
2	Took water from bucket (said to be chlorinated). Boiled some water, didn't wait enough for it to cool *"The water is meant to be cooled after boiling but I did it this way because you said you wanted to see me making it".* Shook some of the ORS sachet into large plastic cup, tasting and adding more several times. Then washed container (large plastic bottle with lid) she will store ORS in & will cover it. Jar leaks so put on dish.	Yes	No	No	Yes	No
3	Used boiling water and added sachet directly, didn’t add everything but folded over sachet and kept remaining, didn’t measure water at all, put it in a plastic jug (not 1 L).	Yes	No	No	No	No
4	Started to make by boiling water on stove (was taught by clinic a long time ago – 1 year), then power went off and she just made in a jug with normal water they have (not treated). Used 3 large plastic cups to measure 1 L (we checked, was correct) into large saucepan. Used whole sachet. Said will clean milk container very well with soap and will store it there. Said they usually boil water on a charcoal fire but hadn’t started fire yet as she was at the clinic which is why used stove. Will keep ORS until the next day. Will cover ORS.	No	Yes	Yes	Yes? (Not observed)	No
5	First mentioned need to start brazier to boil water, then mentioned that stored water was chlorinated so agreed to make the ORS using this water. Washed a spoon, poured some of sachet contents into a large plastic cup of the chlorinated water.	Yes	No	No	Not while researcher was present	No
6	Boiled water on a brazier inside house. Waited for water in saucepan to cool. Put entire contents of sachet into one banana cup (500 ml) of the boiled water.	Yes	No	Yes	Not while researcher was present	No
7	Says uses "own initiative to boil water first". Boiled *some* water in a saucepan. Said she would wait for water to cool. As volume was already incorrect (and ORS would need remaking), researcher asked her to go ahead and prepare the ORS. She added the whole sachet of ORS to the water in the saucepan and stirred with spoon.	Yes	No	Yes	Not while researcher was present	No
8	Uses water purifier sachets to clean water. Four sachets seen in baby bag. Water taken from bucket in kitchen using metal tea pot. Mother has 2 L and 2.5 L containers only. After making ORS mother observes volume is too much. Mixes whole sachet. Keeps ORS in covered metal pot.	Yes	No	Yes	Yes	No
NGWERERE						
1	Makes fire on brazier with charcoal (takes about 5 mins to light, then a long time to heat before can add saucepan of water). Did not observe measurement so must have been before boiling - Mother reported using 1 L sour milk container to measure when questioned. Puts whole sachet of ORS into saucepan but doesn't stir. Covers with lid.	Yes	Yes (measured after boiling)	Yes	Yes	Yes
2	Sent someone to go and buy charcoal and then lit fire, took a long time for water to boil. Poured from saucepan into a jug with a lid. Didn’t measure water at any point. Was about to pour in sachet to complete when we stopped her [so as not to waste the sachet]. Knows how to measure one litre: 3 ½ cups (we checked)	Yes	No	Yes	Not while researcher was present	No
3	15 years old brother-in-law made fire with charcoal which used to boil water. Father went to buy charcoal from nearby market. Mother measures 5 glasses of water (about 1 l) into saucepan and then boils water in large pot. Pours water into plastic jug and covers with lid. Leaves to cool. Adds sachet (not observed)	Yes	Yes (measured after boiling)	Yes	Yes	Yes
4	Boiled water. Cooled in saucepan placed in bucket of cold water. Poured water from saucepan into plastic kettle (2 L capacity). Believes measurement is 2 L. Adds whole sachet and stirs. Puts lid on kettle.	Yes	No	Yes	Yes	No
5	Took a while to light brazier and heat coals. Measured 3 large plastic cups of water into a kettle and boiled water. Rinsed a bowl and poured boiling water into it. Left to cool. Added part of sachet only.	Yes	Yes (measured after boiling)	No	Not while researcher was present	No
6	Is very quick. Mother has firewood already burning, water boils v quickly. Pours from saucepan into 1 l large cup and transferred to larger jug and left to cool (covered). Mixed in whole sachet and mixed by pouring between two containers. Will keep in jug with a lid.	Yes	Yes	Yes	Yes	Yes

Only four of 14 participants prepared ORS correctly, i.e. measured a litre of cool clean water and mixed in a whole sachet of ORS. All caregivers used or attempted to use water that was either boiled or chlorinated or both. Boiling water for drinking was not normal practice, and it took substantial time and effort, sometimes more than an hour, to accomplish this. In two cases someone had to first fetch charcoal from the local market. The quantity of water was measured correctly by six caregivers, although three of them measured before boiling the water which is not recommended. The remainder either did not measure the water at all (*n* = 5) or measured an incorrect amount (*n* = 3), so that the resulting solution was typically too concentrated. In four instances the caregiver used only part of a sachet, mixing it in until satisfied with the taste.

### Factors influencing Behaviour 2: Incorrect preparation of ORS

#### Factors in the Brain

As far as the executive control of behaviour is concerned, mothers had mixed *knowledge* of ORS. It was described as *‘glucose’* and *‘just* water’ and was classed with ‘rice water’ by focus group participants who saw the two liquids as fulfilling a similar function in rehydrating a child. ORS was not viewed as a medicine and there was no *knowledge* that the wrong concentration of the prepared solution could adversely affect the child. Women who were more familiar with preparing homemade sugar-salt solution added and mixed ORS sachet contents using taste as a *cue* that they had prepared the correct concentration.

Knowledge about use of clean water was high, but knowledge on the need to measure 1 l of water was poorer.

Mothers preferred to make up smaller quantities than the recommended litre, so as not to waste any solution that the child would refuse to drink (the *hoard* motive).

*‘The child does not finish drinking 1 litre… It is very wasteful for me to make so much which I end up pouring away. This is why I have opted to make it in a cup so I can save the remainder to make later if needs be’ (Mother, Peri-urban).*

At follow up visits caregivers were keen to demonstrate their ORS preparation skills and volunteered that they would be happy to teach others the same (*affiliation* motive).

#### Factors in the Environment

The physical environment challenged ORS preparation in several ways. Making ORS required a number of objects (brazier, charcoal, saucepans, a vessel to measure water, a container and lid to store the ORS to prevent contamination, etc.), many of which were borrowed from neighbours. Water for ORS was measured using different sized cups and containers, none of which were standard across households and most of which were of unknown capacity. Some caregivers were familiar with earlier teaching to use the ‘banana cup’ (500 ml) to measure out water, but these cups were no longer in common use.

### Behaviour 3: Trial of zinc for diarrhoea treatment

Zinc was not used for diarrhoea treatment by this population prior to our study. No behaviour trial participants and only two focus group participants reported having used zinc on a previous occasion.

Zinc was administered daily throughout the 10-day trial by almost all participants, as requested. They invariably dissolved the tablet in water on a spoon or in a cup before giving it to the child and the tablet was halved to give the correct dosage for the child under six months. Zinc suspension was observed to be spat out by several young children, suggesting that they did not like the taste. One participant discontinued the zinc course at day three of the 10-day course. Nine participants gave the child the full course of tablets, although half of the participants had given the child an incorrect number of tablets by the time of the second (*n* = 4) or third (*n* = 5) visits; in general only one or two tablets were missed.

### Factors influencing Behaviour 3: Trial of zinc for diarrhoea treatment

#### Factors in the Brain

Only one behaviour trial participant had prior *knowledge* of zinc. According to one pharmacy worker, if the medicine prescribed is expensive or unknown to the client (like zinc), they can be reluctant to spend money to try it. Caregivers indicated that they select a course of treatment based on remedies they know and consider effective:

*‘I like this medicine [zinc], it is effective. I have never used it before; this is my first time.... If I was given medicine at the clinic and thought it was not effective, I would resort to using traditional remedies like Mulberry trees and Musiniga trees then give the child to drink’ (Mother, Rural).*

As zinc is not currently used it is not available at home, nor procured at the start of a diarrhoeal episode, nor considered as a treatment option.

The *curiosity* and *nurture* motives probably contributed to the good reception of zinc amongst mothers and their positive reactions to the behaviour trial. They reported being curious to see if zinc could really stop diarrhoea as well as their preferred treatments such as injections and antibiotics which they *believed* had superior effects. In three households zinc tablets were taken by other family members who had diarrhoea and wanted to see if the zinc would stop it. More than one child was reported to dislike the *taste* of zinc, but caregivers indicated that this was the case with other medicines too and did not affect ongoing use during the behaviour trial:

*‘You can’t stop when the child refuses, you just have to continue persuading the child until he finally gives in and takes the medicine’ (Mother, Rural).*

*‘I increase the water so that even when he has spat it out, there is still some left over in the stomach. So that’s what I have been doing. I hold him down and give him the medicine. He spits out a little. I did the same today’ (Mother, Rural).*

Two caregivers disguised the tablets by dissolving them in another drink, while several others reported that they had enlisted the help of another family member or the father who had more authority.

At recruitment many participants insisted they would not forget to give zinc daily as the presence of their ill child would act as a *cue* to remind them The trial calendars received a mixed reception: some used it, others had the calendar on the wall but did not own a pencil to check the days off, while others reported the calendar lost or damaged. Some mothers took the initiative to keep zinc in a place that was visited daily but out of the reach of children, such as a laundry basket. This proved a successful reminder, as did setting alarms on mobile phones (when charged).

#### Factors in the Environment

The *physical* form of zinc in generic blister packs and its unfamiliar name caused two caregivers to confuse zinc with paracetamol. Clinic staff reported that they often remove tablets from their packaging and put them into clear, unmarked plastic bags, a further possible source of confusion.

Lack of choice of treatment options, particularly injections to stop diarrhoea in government clinics, was described by a doctor as a reason why those who could afford it used the private sector. This was explored and confirmed in discussion with caregivers:

‘*When I go to the clinic I go with the expectation that they will give the child an injection and sometimes the child is given only panadol [paracetamol]… What will the Panadol do?’ (Focus group participant, Rural).*

Personal experiences and the opinions of others are important *social* factors influencing treatment choices: zinc is simply not known. The limited supply of drugs at clinics and lack of availability in the private sector in rural areas reduces the opportunity for caregivers to use zinc.

In some households zinc tablets were given by more than one adult. This caused confusion and resulted in children missing tablets on multiple days.

## Discussion and conclusions

This formative research study explored behaviour concerning ORS and zinc and its determinants in the home management of childhood diarrhoea in a rural and urban setting in Lusaka Province, Zambia. The study was conducted to inform the design of a behaviour change campaign.

Behaviour regarding ORS use was suboptimal: no participants used it for the full duration of the diarrhoeal episode and only four prepared ORS solution correctly. Most concerning was the time taken to initiate oral rehydration therapy and the frequent preparation of overly-concentrated ORS solution, as both actions can affect the ability of ORS to replace fluids and correct electrolyte imbalances [5, 24].

Despite caregivers’ reports to the contrary, ORS administration at the outset of a diarrhoeal episode is slow. Diarrhoea is common and a clinic visit takes time and is delayed. This pattern of behaviour in care-seeking is consistent with other studies [25–27]. However, as ORS sachets are typically obtained at the clinic, early initiation of ORS is also delayed. Avoiding a clinic visit may also avoid speculation in a close-knit community about the child’s illness and possible HIV status; ‘disease stigmatisation’ [28] is documented in Zambia [29–31] and elsewhere [32]. Once at a clinic, waiting times and lack of staff available to supervise ORS use at the ORT corners further discourages early use. The final delay in initiation of ORS comes during its preparation: boiling water is cumbersome and may be delayed or avoided altogether. A study in rural China found that ORS use was positively correlated with habitually boiling drinking water [33]. Once a child looks better there is little incentive to continue to prepare new ORS solution. The low number of ORS sachets provided per person at the clinic does not help to reinforce the recommendation that ORS should be given daily while diarrhoea persists.

When caregivers prepare ORS solution that is too concentrated it is because they mix all or part of the sachet contents into an insufficient quantity of water, either to avoid wastage of solution or salts or while making ORS to taste. Manufactured ORS has been shown to be more effective and safer than homemade sugar-salt solutions [5]. It is also generally considered safer because of consistent errors in the measurement of sugar and salt for homemade ORS [21]. Change over time in the messages accompanying co-promotion of both forms of ORS has inadvertently caused confusion. Using only part of a sachet to conserve – or *hoard -* the rest is a natural human tendency. Evolutionary biology tells us that such a motive was adaptive for ancestors who lived in resource-scarce environments [34]. This practice is not unique to this study setting [35]. Hoarding ORS may also be connected with the number of sachets provided by a clinic; availability of ORS at clinics and use of ORS are closely related [33, 36].

There was almost universal lack of awareness of zinc as a diarrhoea treatment, which reflects the limited supply of zinc to government clinics and absence of published studies on zinc promotion in Zambia. Nevertheless, caregivers complied well with recommended behaviour concerning zinc use. Zinc was well-accepted because it was received at the clinic and couched as a medicine that stops diarrhoea, thus meeting the needs of caregivers who were actively seeking treatment. Our finding that caregivers strongly believe in the efficacy of injections and antibiotics is not new [37–39]; if caregivers find that zinc does indeed limit the length of episodes, the widespread introduction of zinc use may help to counter these expectations.

These formative research findings are important because of their implications for intervention design. Encouraging caregivers to give ORS solution early and to continue throughout a diarrhoeal episode to prevent dehydration, rather than just treat its symptoms is challenging: it is hard to motivate people to do something when the reward is not immediate, or obvious. We need to find ways to make such behaviour more rewarding. *Nurture* – caring for a growing child - is an important and essential motive that affects child development [15, 40]. The nurture motive is likely to be a strong driver of good ORS practice for the duration of marked sickness, but not for prolonged correct use. As mothers wish to avoid social judgements about their child’s health or their childcare abilities, an intervention could also explore use of *affiliation* or *status* motives [34, 41] as emotional drivers of behaviour change.

There are differences of opinion about the role of knowledge as a driver of behaviour; campaigns based on health education are often ineffective at instigating change in the target behaviour [42]. However, as skill levels affect a person’s capacity to perform certain behaviours, improving caregivers’ ability to prepare ORS through intervention could be important. ‘Action knowledge’ [43] such as this is a prerequisite for behaviour [44, 45]. In this case, lack of knowledge was not the only cause of incorrect ORS preparation, as mothers sought to economise by using only part of a sachet, leading to measurement error. Changing the design of the ORS sachets could improve correct preparation without an explicit intervention to change behaviour. Several investigators, including the Diarrhoea Working Group at the UN Commission (through CHAI and PATH) and ColaLife in Zambia have explored the potential market for smaller sachets. The latter organisation’s ‘*Kit Yamoyo’* has demonstrated that use of the kit’s packaging can improve correct preparation and directly address measurement challenges [46]. In August 2015 the Government of the Republic of Zambia started distribution of co-packaged 200 ml ORS and zinc and similar products are becoming available in the private sector through a large supermarket chain. Innovation in product design is an important long-term solution, but there is still a clear need to focus efforts on improving preparation of the more widely available and distributed ORS sachets. At the same time it might make sense to revisit the requirement to boil water.

What mothers want most is a powerful way to stop the diarrhoea that their child is experiencing. As zinc offers this, the curiosity factor may be sufficient to drive initial use of zinc. Zinc trials in several countries have demonstrated that promotion of zinc can enhance ORS uptake and reduce use of antimicrobials and antidiarrhoeals [47–51]. This suggests that zinc has the potential to replace Flagyl™ and other antibiotics as the preferred drug for diarrhoea treatment if awareness increases, the population becomes convinced of its effectiveness and supply is assured.

To our knowledge, ORS preparation has not been observed using video cameras in other settings, an aspect of the research that generated rich data on actual practices and barriers. There is a possibility that filming influenced behaviour and that prior notice of visits to the home caused participants to prepare ORS solution that they would not have otherwise made. Further, clinic recruitment of participants for the behaviour trials may have resulted in a sample that was more receptive to zinc and knowledgeable about ORS than the typical population. The total sample size was small, but data were collected iteratively and there was little heterogeneity in practice or response. If clinic presentation and ORS and zinc use are associated with perceived diarrhoea severity and type of diarrhoea (i.e. diarrhoea not caused by teething) [52], use of ORS and zinc in the wider population may be less common than found here.

Theory has an important role to play in intervention design and evaluation, in particular so that generalisable lessons can be taken from particular contexts [53–56]. The ‘Evo-Eco’ theoretical framework from the BCD approach proved useful for structuring research methods to investigate hypothesised determinants of these behaviours and to organise study findings. Insights from the formative research will be used to develop an intervention to improve diarrhoea treatment and control following the BCD approach [14].

### Ethics approval and consent to participate

Ethical approval for the study was granted by the London School of Hygiene and Tropical Medicine Ethics Board (Ref 6286) and the University of Zambia Biomedical Research Ethics Committee (Ref 012-10-12). Voluntary written informed consent was obtained from all participants, including for the use of video and photos taken during ORS preparation.

### Consent for publication

Not applicable.

### Availability of data and materials

Given this was a qualitative formative research study, the data consists almost entirely of videos and interview/FGD transcripts and therefore, to ensure participant confidentiality, we cannot make the data available.

## Abbreviations

BCD: Behaviour Centred Design
ORS: Oral Rehydration Salts
PAED: Programme for Awareness & Elimination of Diarrhoea
